# Palbociclib in combination with aromatase inhibitors in patients ≥ 75 years with oestrogen receptor-positive, human epidermal growth factor receptor 2 negative advanced breast cancer: A real-world multicentre UK study

**DOI:** 10.1016/j.breast.2021.10.010

**Published:** 2021-10-28

**Authors:** Salma El Badri, Bilal Tahir, Kirsty Balachandran, Pavel Bezecny, Fiona Britton, Mark Davies, Karen Desouza, Simon Dixon, Daniel Hills, Maung Moe, Thomas Pigott, Andrew Proctor, Yatri Shah, Richard Simcock, Anna Stansfeld, Alicja Synowiec, Marianna Theodoulou, Mark Verrill, Anshu Wadhawan, Catherine Harper-Wynne, Caroline Wilson

**Affiliations:** a Weston Park Hospital, Whitham Rd, Broomhall, Sheffield, S10 2SJ, UK; b Department of Oncology and Metabolism, The University of Sheffield, Beech Hill Road, Sheffield, S10 2SF, UK; c Charing Cross Hospital, Imperial College Healthcare NHS Trust, Fulham Palace Rd, London, W6 8RF, UK; d Blackpool Victoria Hospital, Whinney Heys Rd, Blackpool, FY3 8NR, UK; e The Christie NHS Foundation Trust, Ogelsby Cancer Research Centre, Manchester, M20 4GJ, UK; f Singleton Hospital, Sketty Ln, Sketty, Swansea, SA2 8QA, UK; g Nottingham City Hospital, Nottingham University Hospitals NHS Trust, Hucknall Rd, Nottingham, NG5 1PB, UK; h School of Health and Related Research, The University of Sheffield, 30 Regent Street, Sheffield, S1 4DA, UK; i Leeds Cancer Centre, St James's University Hospital, Beckett St, Leeds, LS9 7TF, UK; j York Teaching Hospitals NHS Trust, Wigginton Rd, York, YO31 8HE, UK; k Mount Vernon Cancer Centre, Rickmansworth Rd, Northwood, HA6 2RN, UK; l Sussex Cancer Centre, University Hospitals Sussex, Eastern Rd, Brighton, BN2 5BE, UK; m Freeman Hospital, Freeman Rd, High Heaton, Newcastle upon Tyne, NE7 7DN, UK; n Kent Oncology Centre, Maidstone and Tunbridge Wells NHS Trust, Hermitage Ln, Maidstone, ME16 9QQ, UK; o Royal Preston Hospital, Sharoe Green Ln, Fulwood, Preston, PR2 9HT, UK; p Velindre University NHS Trust, Velindre Rd, Whitchurch, Cardiff, CF10 2TL, UK

**Keywords:** Palbociclib, Breast cancer, Real-world, Frail elderly, Toxicity, Treatment efficacy, Cost analysis

## Abstract

**Background:**

Breast cancer incidence increases with age and real-world data is essential to guide prescribing practices in the older population. The aim of this study was to collect large scale real-world data on tolerability and efficacy of palbociclib + AI in the first line treatment of ER+/HER2-advanced breast cancer in those aged ≥75 years.

**Methods:**

14 cancer centres participated in this national UK retrospective study. Patients aged ≥75 years treated with palbociclib + AI in the first line setting were identified. Data included baseline demographics, disease characteristics, toxicities, dose reductions and delays, treatment response and survival data. Multivariable Cox regression was used to assess independent predictors of PFS, OS and toxicities.

**Results:**

276 patients met the eligibility criteria. The incidence of febrile neutropenia was low (2.2%). The clinical benefit rate was 87%. 50.7% of patients had dose reductions and 59.3% had dose delays. The 12- and 24- month PFS rates were 75.9% and 64.9%, respectively. The 12- and 24- month OS rates were 85.1% and 74.0%, respectively. Multivariable analysis identified PS, Age-adjusted Charlson Comorbidity Index (ACCI) and number of metastatic sites to be independent predictors of PFS. Dose reductions and delays were not associated with adverse survival outcomes. Baseline ACCI was an independent predictor of development and severity of neutropenia.

**Conclusion:**

Palbociclib is an effective therapy in the real-world older population and is well-tolerated with low levels of clinically significant toxicities. The use of geriatric and frailty assessments can help guide decision making in these patients.

## Highlights

• Palbociclib is a safe and effective treatment in patients ≥75 years.
• Higher rates of treatment discontinuation and dose reductions were noted.
• Dose delays and reductions were not associated with a worse survival outcome.
• ACCI is a predictor of PFS and also for development and severity of neutropenia.

## 1 Background

Breast cancer is the most common cancer in the UK and accounts for 21% of all cancer diagnoses in females aged ≥75 years [1]. The introduction of cyclin-dependent kinase (CDK) 4/6 inhibitors into routine clinical practice has changed the paradigm of management of oestrogen receptor-positive (ER+), human epidermal growth factor receptor 2 negative (HER2-) advanced breast cancer. However, with the older population representing a growing proportion of these patients, it is important to carefully evaluate the available evidence in this group, considering they are under-represented in clinical trials. Those older patients included in trials tend to be highly selected with a lower risk profile compared to the real-world population, with that disparity increasing with increasing age [2,3]. It is, therefore, necessary to collate real-world data to fill the gaps in knowledge, consolidate the evidence and inform clinicians who prescribe CDK4/6 inhibitors to these older patients.

Three CDK4/6 inhibitors are currently approved in the UK and are being used in clinical practice in first line with an aromatase inhibitor (AI) and second line with fulvestrant: palbociclib, abemaciclib and ribociclib. Although these agents have some overlapping toxicities, specific side effects are unique to, or more frequently encountered with, certain agents. This makes it difficult to group them together under one umbrella when analysing safety or efficacy data. Given that palbociclib was the first agent to be approved and is one of the most frequently prescribed drugs in its class in the UK, it was selected as the agent of choice for this study.

Palbociclib is an orally active and highly selective reversible inhibitor of CDK 4 and 6 that blocks the transition from G1 to S phase of the cell cycle [4]. It was available for use in the UK from September 2017 on the patient access scheme and approved by the National Institute for Health and Care Excellence (NICE) in December 2017 for use in combination with an AI for the treatment of ER+/HER2-advanced breast cancer in the first-line setting [5]. This approval followed on from the results of the PALOMA-2 study which showed a significant improvement in progression-free survival (PFS) with palbociclib combined with letrozole compared with letrozole alone [6]. Out of the 444 patients included in the PALOMA-2 trial, only 48 were aged ≥75 years.

A pooled analysis from the PALOMA studies included 83 patients aged ≥75 years who received palbociclib across the 3 trials and it was found to be a safe and effective treatment in this population but noted higher rates of myelosuppression among patients aged ≥75 years [7]. Another pooled analysis of phase III randomised controlled trials examined the efficacy and safety of different CDK 4/6 inhibitors in combination with an AI in 198 women aged ≥75 years which showed higher rates of toxicity and dose modifications. There was a decrease from baseline in quality-of-life measures in women irrespective of treatment with either combination therapy or single agent AI, suggesting no significant impact of CDK4/6 inhibitors on quality of life [8]. The authors recognised the limitations of the differential toxicity profiles of the three CDK4/6 inhibitors.

Real-world studies have looked at the efficacy and safety of palbociclib, some of which focussed on the older patient group. A French real-world study of palbociclib in patients aged over 70 years (PALOMAGE) reported a lower incidence of grade 3/4 adverse events in those aged ≥80 years [9]. A US retrospective analysis included 92 patients aged ≥70 years treated with palbociclib and reported significantly higher rates of dose reductions and delays in patients ≥70, but with no detriment to survival outcomes [10]. Another US retrospective study showed that a lower starting dose was more common is those aged ≥75 years and linked this to worse survival outcomes [11].

The in-patient secondary care cost burden, i.e. the costs associated with hospital admissions, is another important consideration in these patients given their frailty and higher comorbidity burden. The rate of hospitalisation for chemotherapy-related toxicities in older patients with breast cancer increases with increasing comorbidity score [12], and it is important to look at how this compares to palbociclib-related hospitalisation rates and costs in this population.

This national UK study was designed with the aim of collecting a significantly large scale real-world dataset of patients aged ≥75 years to evaluate the tolerability, efficacy and secondary care in-patient burden in the National Health Service (NHS) when palbociclib is used in combination with an AI in the first-line treatment of elderly patients with ER+/HER2-advanced breast cancer. ACCI was used to assess the baseline comorbidity burden in this population and its impact on treatment and tolerability. This study will add to the existing body of evidence from clinical trials and smaller real-world datasets which will help guide prescribing practices and enable clinicians to access data relevant to the patient populations they are treating.

## 2 Methods

### 2.1 Study design

This was a national multicentre retrospective study open to sites across the UK with 14 cancer centres participating. The study protocol was approved by the Clinical Effectiveness Unit at Sheffield Teaching Hospitals NHS Trust, with the required approvals for data storage and transfer obtained from the Information Governance department. The project leads in Sheffield designed an Excel spreadsheet and disseminated it to centres for data collection. Data was collected from medical records and systemic anti-cancer therapy prescribing systems/pharmacy records. Data was transferred in a pseudonymised state. The participating centres were required to register the study in their respective hospital trusts and follow the local policies for data storage and transfer.

### 2.2 Study population

Patients aged ≥75 years with a confirmed diagnosis of ER+/HER2-advanced breast cancer who have received at least one cycle of palbociclib in combination with an AI starting from the first availability of Palbociclib in the UK on clinical trials (March 2016) to data close in Feb 2021 including patient treated on early access to medicines schemes and standard NHS England funding through the Cancer Drugs Fund. The 5 year period for data collection allowed sufficient time for follow-up.

### 2.3 Data source

Data extracted from medical records included age, Eastern Cooperative Oncology Group (ECOG) performance status (PS), concurrent regular medications, co-morbidities, weight, height, total ER Allred score, de novo metastatic disease or recurrent disease, metastatic disease burden, number of in-patient admissions during palbociclib treatment, duration of in-patient admission and investigations performed, best radiological response to palbociclib, reason and date of treatment discontinuation, date of disease progression and date of death, where applicable.

ACCI has been used to assess the baseline comorbidity burden in this population, which is calculated using weighted scores for a range of comorbidities, adjusted for age. The comorbidities include myocardial infarction, congestive heart failure, peripheral vascular disease, cerebrovascular accident or transient ischaemic attack, dementia, chronic obstructive pulmonary disease, connective tissue disease, peptic ulcer disease, liver disease, diabetes mellitus, hemiplegia, chronic kidney disease, leukaemia, lymphoma, acquired immunodeficiency syndrome and solid organ malignancy [13].

Data was also extracted from systemic anti-cancer therapy (SACT) prescribing systems/pharmacy records at each cancer centre. This included method of access to palbociclib, type of AI used, date of start of palbociclib, starting dose, number of cycles received, number of dose modifications, number of dose delays and adverse events.

### 2.4 Outcome measures

The tolerability was assessed using the rates of dose reduction, dose delay and adverse events; the latter categorized using Common Terminology Criteria for Adverse Events (CTCAE v5.0). The reported adverse events during treatment with palbociclib were classed as treatment-related; however, it is worth noting that causality is difficult to ascertain in a retrospective study. For instance, transaminitis resulting from progression of metastatic liver disease may not be clearly distinguished from that related to treatment, except that it is usually reversible if drug-related, and so causality was defined by the clinical teams following patient assessment and subsequent follow up.

Response measures included best radiological response and the clinical benefit rate; defined as the rate of complete response, partial response or stable disease for ≥24 weeks. Exploratory survival analysis included PFS; measured from the date of starting palbociclib until disease progression, death or data censoring, whichever occurred first, and overall survival (OS); measured from the date of starting palbociclib until death or data censoring if still alive. Given the variability in the timing of response assessments in the real world, the response measures and PFS analysis are as close an estimate as could be derived from a retrospective real-world analysis.

In-patient secondary care burden due to adverse events was calculated by using costs per day for non-elective inpatient admissions relating to the relevant healthcare related group (HRG) within the National Reference Cost collection. Where there were multiple HRG groups relating to a particular type of adverse event, an activity weighted mean cost per day was calculated [14]. The cost of X-rays and blood tests was derived from direct access services. The cost of a CT is based on the National Reference Cost for a scan of three areas, with contrast (RD26Z, taken as an activity weighted average across direct access, outpatients and ‘other').

### 2.5 Statistical analysis

Descriptive and inferential statistical analyses were performed. Descriptive statistics included tabular summaries of data. Continuous variables were reported as median (range). Categorical variables were reported as absolute numbers (percentages). Median, 12- and 24-month PFS and OS time-to-event outcomes were calculated using Kaplan-Meier estimates.

Multivariable regression models via the forced-entry procedure were conducted to assess which baseline variables, selected on the basis of clinical expertise, were independent predictors of outcomes. Specifically, multivariable Cox proportional hazards regression analyses were performed to determine the associations of age, PS (0–1 or ≥2), starting dose (125 mg or <125 mg), dose reduction (yes or no), dose delay (yes or no), baseline ACCI and the number of metastatic sites with PFS and OS. Four cumulative odds ordinal logistic regression models with proportional odds were run to determine the effects of age, PS, baseline ACCI and number of metastatic sites on the onset and severity of neutropenia, thrombocytopenia, anaemia and fatigue.

Patients with missing data for a covariate or outcome measure were excluded from the analysis of that outcome measure. Analyses were performed using SPSS (version 26.0, IBM Corporation, Armonk, NY, USA) or STATA (version 16.1, StataCorp LLC, College Station, TX, USA) statistical software. All *P*-values were 2-sided and a *P*-value of <0.05 was considered statistically significant.

## 3 Results

Fourteen cancer centres from across the UK participated in this study with data collection completed in February 2021. 276 patients met the eligibility criteria. Median follow-up time from starting palbociclib was 23.1 months. The baseline demographics are summarised in Table 1. The median age of patients was 78 (range 75–92) years. 79% of patients had an ECOG PS of 0–1 and 19.6% had a PS of ≥2. ACCI scores were calculated using age and recorded comorbidities with higher score reflecting higher comorbidity burden. 53.1% of patients had visceral metastases and 33.7% had bone-only disease. 88% of patients started palbociclib at the standard dose of 125 mg.

**Table 1:** Baseline demographics and clinical characteristics.

Demographic	N (%)
**Median Age (range) – years**	78 (75–92)
**ECOG PS**	
0	65 (23.6)
1	153 (55.4)
2	51 (18.5)
3	2 (0.7)
4	1 (0.4)
Not recorded	4 (1.4)
**ACCI**	
<10	186 (67.4)
>10	87 (31.5)
Not recorded	3 (1.1)
**BMI**	
underweight <18.5	4 (1.4)
normal 18.5–24.9	78 (28.2)
overweight 25–29.9	77 (27.9)
obesity ≥30	56 (20.3)
Not recorded	61 (22.1)
**ER status (Allred score)**	
strongly-positive (score 6–8)	257 (93.1)
weakly-positive (score 3–5)	7 (2.5)
Not Recorded	12 (4.3)
**Burden of metastatic disease**	
visceral metastases – Yes	147 (53.1)
- No	127 (45.8)
Bone metastases – Yes	180 (65.0)
- No	95 (34.3)
Bone only metastases	93 (33.7)
Bone + visceral metastases	85 (30.8)
**No. of organs involved with metastases**	
0	15 (5.4)
1	109 (39.4)
2	89 (32.1)
3 or more	59 (21.7)
**Presentation**	
De Novo metastatic disease	119 (43.1)
Recurrence	156 (56.5)
Not recorded	1 (0.4)
**AI used with palbociclib**	
letrozole	246 (89.1)
anastrozole	18 (6.5)
exemestane	10 (3.6)
Not recorded	2 (0.7)
**Starting dose of palbociclib**	
125 mg	243 (88.0)
100 mg	29 (10.5)
75 mg	3 (1.1)
Not recorded	1 (0.4)

Abbreviations: ECOG = Eastern Cooperative Oncology Group; PS = performance status; ACCI = Age-adjusted Charlson Comorbidity Index; BMI = body mass index; ER = oestrogen receptor; AI = aromatase inhibitor.

The median duration of treatment with palbociclib and AI was 15.7 months (range 1–43.4 months). Treatment was discontinued in 138 patients (50%); this was due to disease progression in 65 (47.1%) patients, toxicity in 36 (26.1%) and death in 22 (15.9%) patients. At least 54 patients had treatment interrupted due to the perceived risks during the COVID-19 pandemic; 35 patients have subsequently restarted treatment. As data collection was started prior to the pandemic, data on treatment interruption was not specifically collected and these numbers are derived from additional notes provided by centres.

The most common toxicities were neutropenia, followed by fatigue, anaemia and thrombocytopenia. Only six patients (2.2%) had febrile neutropenia. Table 2 shows the common toxicities reported with palbociclib in this patient group using the CTCAE v5.0 grading system.

**Table 2:** Treatment-related adverse events – as per CTCAE v5.0

	All grades - n (%)	Grade ≥3 - n (%)
Neutropenia	223 (80.8)	128 (46.4)
Fatigue	148 (53.6)	10 (3.6)
Anaemia	125 (45.3)	8 (2.9)
Thrombocytopenia	102 (37.0)	7 (2.5)
Nausea	61 (22.1)	4 (1.4)
Stomatitis	59 (21.4)	1 (0.4)
Anorexia	53 (19.2)	0
Diarrhoea	51 (18.5)	3 (1.1)
Transaminitis	32 (11.6)	4 (1.4)
Vomiting	24 (8.7)	1 (0.4)

140 patients (50.7%) required a dose reduction, with the majority of these occurring in the first 3 cycles (57.9%). The most common reasons for dose reduction were neutropenia (54.3%), fatigue (21.4%) and thrombocytopenia (5%). 163 patients (59.3%) required at least one dose delay. The most common reasons for dose delay were neutropenia (66.3%), infection (10.4%) and fatigue (6.7%).

Out of the 276 patients, 22 (9.6%) required hospital admission due to toxicity. The median duration of in-patient stay was 6 days (range 1–76 days) and the mean was 11 days (SD: 17 days). The average annual cost per patient for hospital admission due to toxicity was £342.16. Given the small sample size and degree of imputation in calculating cost, this is an estimated average. The calculation incorporated the cost of hospital admissions, blood tests and imaging. The precise toxicity necessitating admission to hospital was specified for 11 patients; 7 of these were due to infections.

**Fig. 1:**
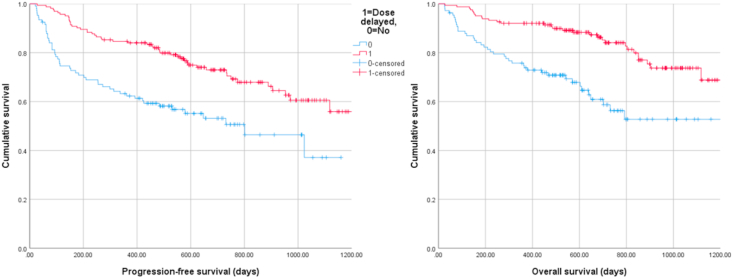
Kaplan-Meier survival plots demonstrating the association between dose delays and progression-free (left) and overall (right) survival.

The best radiological response was complete response in five patients (2.0%), partial response in 81 (32.9%) and stable disease in 135 (54.9%), with 25 patients (10.2%) having progressive disease. Thirty patients did not have a recorded radiological response assessment. The clinical benefit rate at 24 weeks was 87%. The PFS rates at 12 and 24 months were 75.9% and 64.9%, respectively. The OS rates at 12 and 24 months were 85.1% and 74.0%, respectively. Kaplan-Meier curves for PFS and OS were plotted with patients grouped based on whether they experienced a dose delay or not (Fig. 1); log-rank tests demonstrated significant differences in survival distributions for both PFS and OS (p < 0.001).

Multivariable Cox regression identified increased PS, fewer dose delays, increased ACCI and higher number of metastatic sites to be independent adverse predictors of PFS. Significant adverse predictors of OS were increased PS, fewer dose reductions, fewer dose delays, and increased number of metastatic sites. A summary of multivariable Cox regression results for PFS and OS, including hazard ratios (HRs) and 95% confidence intervals (CIs), are displayed in Table 3.

**Table 3:** Summary of multivariable Cox regression results for PFS and OS. HR (95% CI) results for statistically significant predictors are in bold.

Outcome measure	PFS		OS	
	HR (95% CI)	*p*-Value	HR (95% CI)	*p*-Value
Age (continuous)	0.975 (0.917–1.036)	0.413	1.015 (0.949–1.086)	0.655
PS (categorical): 0–1 as reference vs 2-4	**3.132 (1.972–4.973)**	< **0.001**	**3.638 (2.159–6.130)**	< **0.001**
Starting dose (categorical): 125 mg as reference vs < 125 mg	1.459 (0.685–3.104)	0.327	1.883 (0.722–4.912)	0.196
Dose reduction (categorical): no as reference vs yes	0.814 (0.507–1.304)	0.391	**0.535 (0.309–0.927)**	**0.026**
Dose delay (categorical): no as reference vs yes	**0.425 (0.269–0.672)**	< **0.001**	**0.393 (0.232–0.665)**	**0.001**
Baseline ACCI (continuous)	**1.260 (1.033–1.536)**	**0.022**	1.236 (0.990–1.543)	.061
Number of metastatic sites (continuous)	**1.491 (1.253–1.775)**	< **0.001**	**1.471 (1.201–1.801)**	**0.001**

Abbreviations: ACCI = Age-adjusted Charlson Comorbidity Index; CI = confidence interval; HR = hazard ratio; PFS = progression-free survival; PS = performance status; OS = overall survival.

For the toxicity analysis, only baseline ACCI was an independent predictor of onset and severity of neutropenia, odds ratio (OR) = 0.760 (95% CI, 0.597 to 0.968). PS was the only independent predictor of the onset and severity of thrombocytopenia and fatigue with the odds of increasing severity for patients with PS = 0–1 being 0.412 (95% CI, 0.228 to 0.748; p = 0.004) and 0.515 (95% CI, 0.289 to 0.917; p = 0.024) times that of patients with PS = 2–4, respectively. No variable was statistically associated with anaemia.

## 4 Discussion

Palbociclib is the first in class CDK4/6 inhibitor approved for use in patients with advanced ER+/HER2-breast cancer. Given the under-representation of the older population in clinical trials, real-world data is crucial to bridge that gap in knowledge. This national multi-centre retrospective UK study provides the largest known dataset of patients aged ≥75 years treated in the NHS with palbociclib + AI in the first line setting, with a total of 276 patients included.

In this study, 19.6% of patients had a PS of 2 or more, in contrast to only 2% of patients included in the PALOMA-2 trial receiving palbociclib. This is more reflective of the broader real-world population, and more so in the older patients. The proportion of elderly patients presenting with de novo metastatic disease in this study is comparable to that seen in other studies of CDK4/6 inhibitors [6,8,[15], [16], [17]]. ACCI was used to assess the comorbidity burden using an established scoring system that includes age and comorbidities with higher scores reflecting higher comorbidity burden. The majority of patients started palbociclib at the standard dose of 125 mg, 29.5% of these patients had an ACCI >10. 32 patients (11.6%) started at a lower dose of 100 mg or 75 mg, 46.9% of these had an ACCI >10. This reflects a tendency to starting at a lower dose for those with a higher comorbidity burden. None of the patients starting at a lower dose had a subsequent dose escalation. Given the small number of patients starting at a lower dose, it was not possible to assess the effect of lower starting dose on efficacy. Recent results from the US real-world study by Patt et al. (n = 813) showed that lower starting doses were more prevalent in patients over 75 years (38.5% vs 17.1%) with resulting lower real-world PFS (18.6 months vs 27.8 months) and treatment responses (40.4% vs 54.0%) [11]. Taken together with our data which suggests subsequent dose reductions and delays do not adversely affect efficacy, finding the maximum tolerated dose early after treatment initiation should be considered in this older population with subsequent dose modifications according to toxicity.

With regards to tolerability in this patient group, neutropenia was the most frequently occurring adverse event with all grade neutropenia occurring in 80.8%. Although we cannot directly compare to trial data, this was broadly similar to that seen in PALOMA-2 (79.5%). Grade ≥3 neutropenia however was less frequent (46.4% vs 66.5%). Febrile neutropenia was again uncommon in the real-world older population (2.2%). There was a higher frequency of fatigue, anaemia, thrombocytopenia and stomatitis in this study as compared to PALOMA-2, whereas nausea, vomiting and diarrhoea were less common. The rate of hospital admission due to toxicity was relatively low (9.6%), again reflecting good overall tolerability in the older population despite their frailty and comorbidity burden. A review of Medicare data reported on the rates of hospitalisation for chemotherapy-related toxicity in older women with breast cancer in the US which were 12.3% and 13.2% for patients with stage III and IV disease, respectively [12]. Direct comparison of these results, however, is not possible, given the different health systems and also the different time periods, taking into consideration the advances in health care and management of toxicities. A more recent US study of the rates of hospitalisation for complications of systemic therapy reported 11.1% of patients with breast cancer required hospital admission but this was not age- or stage-specific [18]. The results of our real-world study reflect good overall tolerability in the older population and are in keeping with the safety analysis from the PALOMAGE French study which showed palbociclib to be well-tolerated in this population with no new safety signals identified [9].

The rate of treatment discontinuation due to toxicity was higher in this real-world population (13%), in contrast to 9.7% in those receiving palbociclib and letrozole in the PALOMA-2 trial. There was also a significantly higher rate of dose reduction (50.7%), compared to only 36% of patients in the trial requiring ≥1 dose reduction. The rate of dose delays was lower compared to the trial (59.3% vs. 67%). Despite the higher rates of treatment discontinuation and dose reductions, the clinical benefit rate at 24 weeks was comparable (87% vs 84.9% in PALOMA-2).

Looking at the in-patient secondary care burden, the hospital admission rate due to toxicity was lower in this study compared to that associated with chemotherapy [12], which reflects the better tolerability of palbociclib. This is especially important in the older population with multiple comorbidities. The average annual cost per patient for palbociclib toxicity-related hospitalisation in our study was £342.16. One other UK study has looked at the costs of managing chemotherapy-associated toxicities in third line metastatic breast cancer patients and showed average annual costs ranging between £2621 and £2740 [19]. However, we are not able to make a valid comparison with the results of that study as that work is only available as a conference abstract and the methods are not described in sufficient detail. Our own study also has limitations which meant that unit costs were not related to specific toxicities and we were restricted to costing only a small number of hospitalisations. Another limitation is the different timelines of the two studies and the possible changes in management of toxicities should be taken into account with a more cost-effective utilisation of resources over time. However, given the importance of cost analysis in the development of treatment guidelines, it is important that future studies of toxicities incorporate this.

The survival results from this study looking at the 12 and 24-month PFS and OS rates were also reassuring. Not surprisingly, higher ACCI and higher metastatic disease burden as defined by number of metastatic sites were associated with worse PFS. Interestingly, and in keeping with results from other analyses, dose reductions and delays were not associated with worse survival outcomes [10]. Dose delays were actually found to be associated with longer PFS and OS in this real-world UK study; dose reductions were associated with longer OS. These results are reassuring to the prescribing clinicians as well as patients that de-escalation of palbociclib dose or frequency does not negatively impact on efficacy; however, the data here cannot claim superiority of disease outcomes with dose delays/reductions due to the limitations of relatively small patient numbers and this being a retrospective study rather than a prospective controlled trial. Considering commonly attributed favourable prognostic characteristics such as bone-only disease and de novo presentation of disease, the group needing dose delays had a similar proportion of bone-only disease compared to those not needing a dose delay (36.6% vs 30.6%, respectively) and the proportion of de novo disease was higher in the group not requiring dose delays (51.4% vs 38.0%). The biological rationale for the effect of dose delays and reductions on survival outcomes is not clear but age-related changes in the pharmacokinetics and pharmacodynamics of drugs, including reduced hepatic metabolism and renal excretion, and changes in cancer biology with a relatively indolent disease in the older population, may all result in efficacious dosing of drugs in these patients that is different to the standard dosing in the younger population [20]. Increasing age does have a statistically significant effect on total clearance of palbociclib from plasma (CL/F) and at an age of 74 years, CL/F was predicted to decrease by approximately 8% compared to the value at 61 years [21]. This may, in itself, not be considered a clinically relevant effect, but when considered in a frailer population with polypharmacy, there could be an effect on concurrent co-morbid conditions affecting all-cause mortality in a population who may die with, rather than of, their breast cancer. Another theory to consider is toxicity as a potential predictive biomarker for response which is observed with other anticancer drugs and would give a plausible explanation for those with dose reductions and delays having better survival outcomes [22]. This data should not change prescribing practice but is certainly hypothesis generating and requires further consideration.

Looking at predictors for toxicity, increasing ACCI was an independent significant predictive factor for onset and severity of neutropenia, which would therefore be important to take into consideration when considering patients suitable for longer follow-up intervals on treatment. Good PS (0–1) was found to be a predictor of worse thrombocytopenia and fatigue which cannot be clearly explained, however one speculation could be that those with worse PS have more dose reductions and delays due to alternative causes, which in turn could result in reduced incidence and severity of thrombocytopenia and fatigue.

This large-scale study has shown palbociclib to be a well-tolerated treatment in older patients with advanced ER+/HER2- breast cancer despite their frailty and comorbidity burden. Efficacy outcomes were also consistent with no detriment to survival outcomes despite higher rates of treatment discontinuation and dose reduction. This is in keeping with the recommendations made by the joint European Society of Breast Cancer Specialists and the International Society of Geriatric Oncology (SIOG) taskforce which recommends CDK4/6 inhibitors as a suitable treatment in older patients given that efficacy is age independent as shown from the pooled analyses of the landmark trials, though higher rates of toxicity and dose modifications were acknowledged in those aged ≥75 years [23].

ACCI proved to be a significant predictor of PFS as well as for development and severity of neutropenia in this study. The authors acknowledge that ACCI only captures the comorbidity domain of geriatric assessments but this was chosen as a surrogate as it is the most consistently documented and readily available data for the purposes of a widescale retrospective review within the NHS. Comprehensive geriatric assessments are recommended by SIOG and the importance of incorporating them in clinical trials and prospective observational studies of CDK4/6 inhibitors was highlighted in a review paper by Battisti et al. [24,25]. The use of ACCI as an independent predictor of survival and toxicity in elderly patients receiving CDK4/6 inhibitors would need to be validated, but the results from this study further reinforce the importance of using baseline geriatric and frailty assessments to aid discussions and monitoring on treatment.

Some of the limitations of this study have already been discussed. Other limitations to acknowledge given the retrospective nature of data collection include the lack of a control group to allow comparative efficacy, lower accuracy and inferior quality of data as compared to clinical trials and the lack of comprehensive geriatric assessments as discussed, although ACCI was used as a surrogate. Another important limitation is the lack of data on quality of life which is a key metric in this older population with limited life expectancy. Nevertheless, this large UK real-world study adds to the existing body of evidence and sheds light on potential predictive factors for response and toxicity with palbociclib in the older population, which would need to be explored further in prospective studies.

## Authors’ contributions

S El Badri collected data and wrote the manuscript. BA Tahir conducted statistical analysis and edited the manuscript. S Dixon contributed to the cost analysis. K Balachandran, P Bezecny, F Britton, M Davies, K Desouza, D Hills, M Moe, T Piggott, A Proctor, Y Shah, R Simcock, A Stansfeld, A Synowiec, M Theodoulou, M Verrill, A Wadhawan and C Harper-Wynne collected data and reviewed final manuscript. C Wilson conceived and designed the project, edited the manuscript and is the senior author.

## Funding information

This research did not receive any specific grant from funding agencies in the public, commercial, or not-for-profit sectors.

## Declaration of competing interest

S El Badri, BA Tahir, K Balachandran, P Bezecny, F Britton, M Davies, S Dixon, D Hills, M Moe, T Pigott, Y Shah, A Stansfeld, A Synowiec, M Theodoulou and A Wadhawan have no conflicts of interest. K Desouza has research grants from Roche and has been an invited speaker for Pfizer. C Harper-Wynne is on the advisory board for Pfizer, Roche, Lilly and Exact sciences and has been an invited speaker for Novartis, Myriad and Veracyte. A Proctor is on the advisory board for GSK and has been an invited speaker for Pfizer and GSK. R Simcock is on the advisory board for Novartis and Exact Sciences and has been an invited speaker for Novartis. M Verrill has received honoraria from Pfizer, Lilly, Novartis, Roche, MSD, Seagen, Daiichi-Sankyo, Exact Sciences and Gilead, and research funding from Seagen, Roche, Novartis, Pfizer, Lilly and Exact Sciences. C Wilson has institutional grants from Pfizer and has been an invited speaker for Pfizer, Novartis, Lilly and Roche.
